# Bubble Hair Deformity Induced by Bathroom Heating Lamp: A Report of Two Cases

**DOI:** 10.1111/jocd.70312

**Published:** 2025-06-26

**Authors:** Daxing Wu, Lifeng Wu, Jianzhong Xu

**Affiliations:** ^1^ Department of Dermatology Tongxiang Dermatology Hospital Tongxiang City Zhejiang Province People's Republic of China; ^2^ Department of Dermatology Tongxiang First People's Hospital Tongxiang City Zhejiang Province People's Republic of China

**Keywords:** hair disorders, hairloss, thermal injury

Dear sir,

Bubble hair deformity was first described by Brown et al. in 1986 [1]. Since then, over a dozen cases have been reported, all of which appear to be linked to prolonged exposure of wet hair to high temperatures from blow dryers or other hair styling devices. The heat causes water within the hair shaft to vaporize, leading to hydrolysis of keratin, local air expansion, and ultimately bubble formation [2]. We hereby report two cases of bubble hair deformity triggered by bathroom heating lamps.

*Case 1*: A 28‐year‐old male presented with hair color changes and thinning in the frontal and crown areas over the past 2 weeks. He reported no symptoms on the scalp itself, no significant medical history, and denied any history of perming or exposure to other chemicals. Upon further inquiry, the patient mentioned using a bathroom heating lamp during showers due to cold weather, during which his scalp was in close proximity to the lamp for prolonged periods. He had experienced a similar episode the previous winter, which resolved spontaneously without treatment. On cutaneous examination, the patient's scalp appeared greasy with increased dandruff. Hair in the frontal and crown areas was sparse, and the hair shafts were dry, yellowed, and twisted (Figure 1A). The hair pull test revealed broken hairs. Dermoscopy showed increased scalp scales and broken hair, with some ends split and broom‐like in appearance (Figure 1B). Small air bubbules were also observed under light microscopy (Figure 1C).

**FIGURE 1:**
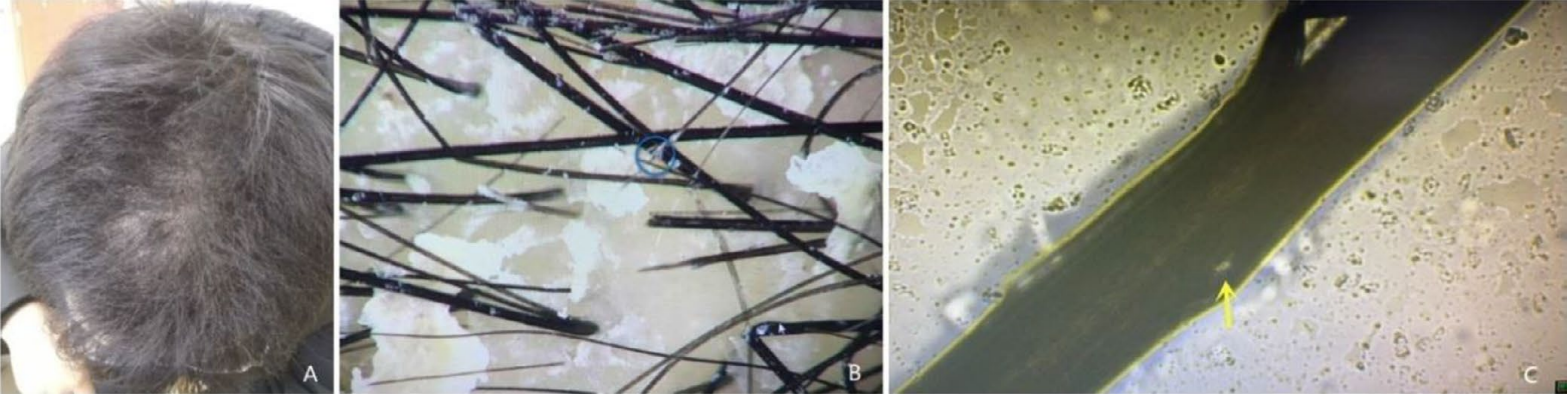
The clinical, dermoscopic, and light microscopic changes of case 1: sparse, dry, and yellowed hair in the frontal and crown areas (A), with broom‐like broken ends observed on dermoscopy (B), and small air bubbles under light microscopy (C).

*Case 2*: A 27‐year‐old male presented with dry, brittle hair on the crown for 1 week. He denied any history of perming or scalp friction but reported recent use of a bathroom heating lamp. Due to the lamp's low installation height, his scalp was in close proximity to the heating light during showers. On the crown, there was a patch about the size of a palm with dry, irregularly broken hair (Figure 2A). The hair pull test showed broken hairs, but no bald patches or significant hair loss in other areas were observed. Dermoscopy revealed angular fractures of the hair shafts, with some nodular incomplete breaks and others completely broken with broom‐like ends (Figure 2B). Splitting and destruction of the hair shaft were observed under light microscopy (Figure 2C). Fungal microscopy was negative.

**FIGURE 2:**
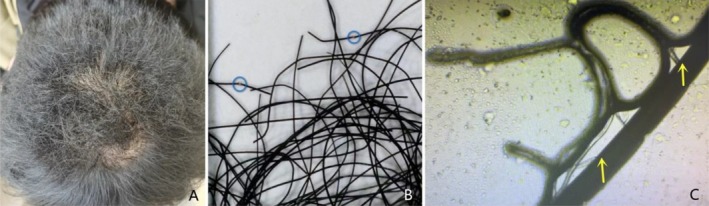
The clinical, dermoscopic, and light microscopic changes of case 2: dry and broken hair on the crown (A), with angular fractures and broken hairs seen on dermoscopy (B), and splitting and destruction of the hair shaft under light microscopy (C).

Based on the medical history, clinical presentation, dermoscopic, and microscopic findings, both cases were diagnosed with bubble hair deformity. The condition was explained to the patients, and they were advised to avoid exposure to heat sources. No specific treatment was administered. During a follow‐up phone call 3 months later, both patients reported that the proximal hair shafts of the bubble hairs had returned to normal. After a haircut, the appearance of the affected area had significantly improved.

Bubble hair deformity is a reproducible disorder of the hair shaft caused by thermal injury [3]. Clinically, it presents with kinked, broken, and discolored hair. The appearance is typically clumped, with a rough and stiff texture, potentially leading to localized hair loss. While the diagnosis has traditionally been made using light microscopy, dermoscopy is a more convenient and efficient tool. However, it primarily reveals dysmorphia of the distal hair shaft [2].

The differential diagnosis for bubble hair deformity includes various acquired and congenital hair shaft abnormalities [4], such as pili annulati and fungal infections, which can be distinguished through a thorough medical history and mycological examination.

Most reported cases of bubble hair deformity have involved Caucasian females, with only one reported case in a Southeast Asian individual [2]. The common triggers for these cases include blow‐drying and other hair styling practices [2]. To our knowledge, this is the first report of bubble hair deformity induced by bathroom heating lamps. In contrast to previous reports, both of our cases involved young males, with the trigger being the proximity to a bathroom heating lamp during showering. Given the widespread use of bathroom heating lamps as heating devices in China, this is an important consideration for both dermatologists and the general public. Maintaining a safe distance from such heating devices is the primary preventive measure.

## Conflicts of Interest

The authors declare no conflicts of interest.

## Data Availability

The authors have nothing to report.
